# Correction: Luminescence property tuning of Yb^3+^–Er^3+^ doped oxysulfide using multiple-band co-excitation

**DOI:** 10.1039/c8ra90037j

**Published:** 2018-05-17

**Authors:** Hong Wang, Xiumei Yin, Mingming Xing, Yao Fu, Ying Tian, Tao Pang, Xin Feng, Tao Jiang, Xixian Luo

**Affiliations:** Physics Department, Dalian Maritime University Dalian Liaoning 116026 PR China luoxixiandl@126.com; College of Science, Huzhou University Huzhou Zhejiang 313000 PR China

## Abstract

Correction for ‘Luminescence property tuning of Yb^3+^–Er^3+^ doped oxysulfide using multiple-band co-excitation’ by Hong Wanga *et al.*, *RSC Adv.*, 2018, **8**, 16557–16565.

In the original publication, the first author’s name was incorrect; the correct version is shown above.

The Royal Society of Chemistry apologises for these errors and any consequent inconvenience to authors and readers.

## Supplementary Material

